# Therapeutic effects of sensory input training on motor function rehabilitation after stroke

**DOI:** 10.1097/MD.0000000000013387

**Published:** 2018-11-30

**Authors:** Xiaowei Chen, Fuqian Liu, Zhaohong Yan, Shihuan Cheng, Xunchan Liu, He Li, Zhenlan Li

**Affiliations:** Department of Physical Medicine and Rehabilitation, The First Hospital of Jilin University, Changchun, Jilin, China.

**Keywords:** motor rehabilitation, sensorimotor integration, stroke

## Abstract

Motor dysfunction is a common and severe complication of stroke that affects the quality of life of these patients. Currently, motor function rehabilitation predominantly focuses on active movement training; nevertheless, the role of sensory input is usually overlooked. Sensory input is very important to motor function. Voluntary functional movement necessitates preparation, execution, and monitoring functions of the central nervous system, while the monitoring needs the participation of the sensory system. Sensory signals affect motor functions by inputting external environment information and intrinsic physiological status as well as by guiding initiation of the motor system. Recent studies focusing on sensory input-based rehabilitation training for post-stroke dyskinesia have demonstrated that sensory function has significant effects on voluntary functional movements. In conclusion, sensory input plays a crucial role in motor function rehabilitation, and the combined sensorimotor training modality is more effective than conventional motor-oriented approaches.

## 1 Introduction

Stroke, whether ischemic or hemorrhagic, is a common cerebrovascular event with high disability and mortality rates. It is the leading contributor to secondary movement disorders in elderly patients.^[1]^ Post-stroke dyskinesia is a common and severe complication that affects the quality of life of these patients. Currently, motor function rehabilitation predominantly focuses on active movement training, such as improving muscle strength, controlling convulsions, and adjusting movement patterns.^[2,3]^ However, rehabilitation training based on sensory input has yet to be highlighted.^[4]^

Voluntary functional movement necessitates preparation, execution, and monitoring functions of the central nervous system; the preparation and execution require involvement of the motor system, while the monitoring needs the participation of the sensory system.^[5]^ In higher-order motor behaviors, the brain must integrate sensory inputs to evaluate the surrounding environment accurately and to produce the corresponding motor outputs.^[6]^ Movement adaptability refers to the ability to adjust constantly to the motor strategy in order to adapt to changes in the environment, which should be based on the feedback of sensory input.^[7]^ Sensory signals affect motor functions in the following 2 ways: inputting external environment information and intrinsic physiological status, and guiding initiation of the motor system.^[8]^

In this review, we summarize the anatomical basis, relevant experimental studies, and clinical applications of sensory input training as well as discuss the therapeutic effects of sensory input training on motor function rehabilitation after stroke. This review highlights the importance of the sensory component of motor function and illuminates the application value of sensory input training for motor function rehabilitation.

## 2 Anatomical basis of sensory input training in motor rehabilitation

### 2.1 Basal ganglia circuit

Sensory afferent nerves directly or indirectly project to the brain stem, cerebellum, subcortex, and cortex. Basal ganglia connect with the frontal lobe, limbic system, and sensory system via the neural circuit; and this circuit participates in the motor control and the integration of cognitive, emotional, and sensorimotor information. Although basal ganglia have no sensory projection fibers, they can govern motor function by processing the sensory information indirectly. Numerous studies have shown that basal ganglia participate in the generation and maintenance of actions in 2 ways: by simultaneously activating the agonistic and antagonistic muscles and maintaining balance, or by sequentially activating the agonistic and antagonistic muscles and generating fast motion.^[9]^ Additionally, basal ganglia can selectively inhibit certain active motions, assisting the body to complete a specific action.^[10]^ Neurophysiological studies have confirmed that basal ganglia are the control center of multi-level sensory input and that abnormal sensorimotor integration is the pathological basis of motor dysfunctions.^[11]^ Among the motor circuit components of the basal ganglia, the substantia nigra, hypothalamus, globus pallidus, and caudate nucleus are the main focus. Moreover, the basal ganglia circuit can be regulated by special dopamine receptors. Sensation-induced phase-related release of dopamine is deemed to be a crucial factor affecting the generation and reinforcement of involuntary movements.^[12]^

### 2.2 Cerebellum circuit

**Figure 1:**
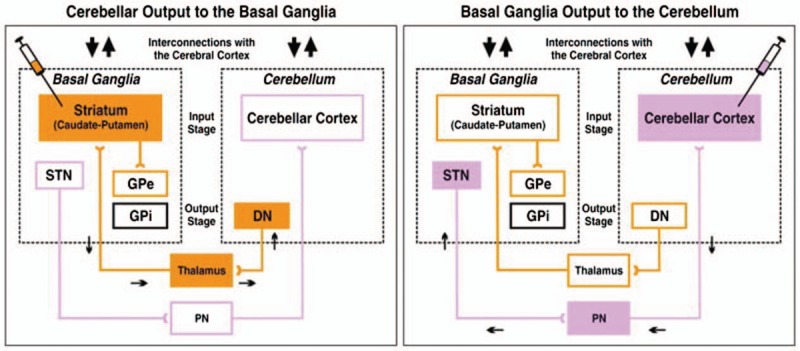
Experimental paradigms and circuits interconnecting the cerebellum and basal ganglia (reference ^[4]^). DN = dentate nucleus, GPe = external segment of the globus pallidus, GPi = internal segment of the globus pallidus, PN = pontine nuclei, STN = subthalamic nucleus.

The cerebellum directly receives abundant sensory afferent fibers, which play an important role in guiding motion and regulating motor coordination.^[13]^ The cortex-cerebellum circuit connects the frontal lobe, pons, cerebellar cortex, deep cerebellar nucleus, locus ruber, ventrolateral nucleus of the thalamus, and motor cortex, which provide an anatomical basis for the regulation of motor coordination. Moreover, the virus tracing technique has shown that dual fiber connections exist among the basal ganglia, sensorimotor cortex, and cerebellum (Fig. 1).^[14,15]^ The cortex–basal ganglia–cerebellum circuit has an essential role in the motor, cognitive, emotional, and sensory functions in patients with dyskinesia.

### 2.3 Sensorimotor center

Previous studies have shown reciprocal fiber projection between the primary motor area (M1) and the primary sensory area (S1).^[16–19]^ The posterior parietal cortex (PPC) is located rostral to the primary and secondary visual cortex, and caudal to the somatosensory cortex; injury in the PPC can cause cognitive, sensory, or motor dysfunction.^[20]^ The PPC receives afferent fibers from 20 cortical areas and 25 thalamic nuclei, and it projects to 25 cortical areas, based on which the PPC participates in the complicated sensorimotor network. Additionally, the PPC is the sensorimotor integration center for active tactile exploratory motions.

## 3 Experimental studies of sensory input training in motor rehabilitation

### 3.1 Animal experiments

A study on mammals has found that sensory input signals by stimulating the skin, muscles, and joints can activate M1 neurons.^[21]^ In addition, Tanji et al have studied the sensorimotor cortex in an unanesthetized monkey; they found that the noncutaneous input activated the caudal part of the M1 and that the cutaneous input primarily activated the caudal part of the M1.^[22]^ Moreover, Xerri et al have demonstrated that the motion control function is impaired in monkeys after neuronal damage in the S1.^[23]^ Damage to the somatosensory cortex usually causes loss of voluntary motor functions and sensation to somatic stimulation. In adult rhesus monkeys, removal of the S1 cortex dominating the distal forearm has been shown to result in severe motor dysfunction and decreased sensation to a tactile stimulus.^[24]^

Experimental studies on rats have revealed that peripheral nerve injury can cause reorganization of the motor cortex.^[25]^ Additionally, Petersen et al have found that whisker muscles are innervated by cholinergic motor neurons located in S1. Stimulation of M1 drives exploratory rhythmic whisking, while stimulation of S1 drives whisker retraction.^[26]^

### 3.2 Experimental studies in humans

Clinical evidence has confirmed the close relationship between sensory function and motor function. Some scholars have noted that a partial or complete loss of sensation impacts the accuracy and coordination of directional movements.^[27,28]^ In a haptically deafferented patient, the loss of sensory input caused a lack of conscious recognition of her own actions.^[29]^ In addition, Kiemel et al have found that light touch can improve postural stability; and they speculated that this may be due to the reinforced consciousness to active movements.^[30]^ Hermsdörfer et al also have noted that the dynamic activation of tactile receptors in the thumb and forefinger guaranteed the stability and accuracy of gripping motions.^[31]^ Furthermore, speech motor outputs are closely correlated with the auditory sensory input.^[32]^

Functional neuroimaging has demonstrated distinct anatomical structures in the M1 area and cerebellum between musicians and nonmusicians.^[33]^ Some studies have proposed that musical training can reinforce the neural connectivity in certain brain areas;^[34–36]^ furthermore, musical activities, such as playing a musical instrument, can improve the neural plasticity, especially in the frontal and temporal regions.^[37,38]^ These findings indicate that the sensory input can help with motor function rehabilitation. Schneider et al have found that music-supported training can improve the motor functions of the upper extremities in post-stroke patients, via strengthening the cortical functional connections and increasing activation of the motor cortex.^[39]^ In addition, Choi et al have used high-frequency repetitive transcranial magnetic stimulation (rTMS) to stimulate the somatosensory cortex, which resulted in improved sensory discrimination ability, muscular synchronized contraction, as well as motor coordination; these findings suggest that rTMS can enhance sensorimotor integration and promote motor rehabilitation.^[40]^

The study design was approved by the Ethics Committee of The First Hospital of Jilin University and written informed consent was obtained from each patient.

## 4 Clinical rehabilitation technologies based on sensory input

### 4.1 Bobath technique

The Bobath concept considers that post-stroke dyskinesia is due to the loss of control of the superior cerebral center to low-level centers and that the inhibition of primitive reflexes is reduced; thus, the Bobath technique advocates the use of a multi-channel sensory input to prevent motor compensation and to remodel the normal motor status.^[41]^ The Bobath technique also emphasizes the role of sensorimotor integration in motor modulation, suggesting that sensory input training is beneficial for motor rehabilitation in post-stroke patients.^[42]^

### 4.2 Proprioceptive neuromuscular facilitation (PNF) approach

PNF refers to a recently advanced form of rehabilitation training involving both the stretching and contraction of targeted muscle groups.^[43]^ This technique is based on human auxology, neurophysiology, and kinesiology. PNF training mobilizes multiple joints and muscle groups, comprehensively using kinesthetics and postural sense to motivate the neuromuscular reaction. This approach modulates the muscular contraction via the proprioceptive sensory system and facilitates motor rehabilitation.^[44]^

### 4.3 Rood technique

The Rood technique, also known as multisensory stimulation therapy, is suitable for all subtypes of motor control deficits. This treatment uses sensory stimulation, such as a fast brush or light touch on skin and tapping on the muscle tendon or belly, to motivate or inhibit the neuromuscular reaction. Additionally, this approach uses squeezing, stretching, or light touch to relieve muscular spasms.^[45]^

### 4.4 Cognitive-motor training

Recently, cognitive-motor training has been extensively used in post-stroke rehabilitation. Relevant studies have found that short-term cognitive-motor training can improve the gait and equilibrium functions in post-stroke patients; however, determining the long-term efficacy still requires further research.^[46]^ Additionally, cognitive-motor training can be employed to predict the risk of falling in elderly patients.^[47]^

### 4.5 Virtual reality (VR) technology

VR rehabilitation is based on the theory that the central processing of postural stability and spatial direction sense rely on multi-sensory input and the requirement for specific motions.^[48]^ VR rehabilitation can provide standardized or individualized intervention on patients’ motor functions in a circumstance with a multi-dimensional sensory input.

### 4.6 Music-based intervention

In recent years, music-based intervention has been widely used in neurorehabilitation, and it has shown remarkable efficacy in improving motor functions.^[49]^ During gait training, rhythmic sound stimulation can significantly improve a patient's walking function, especially in terms of posture control, balance, walking velocity, stride length, standing time, walking rhythm, and symmetry.^[50,51]^ Another study also has observed that music-based rehabilitation significantly improves the motor function of hemiplegic upper limbs.^[39]^ Of note, “mute” musical instruments did not provide an obvious benefit, indicating that the functional improvement was associated with the music sensory input.^[52]^ In addition, Altenmüller et al have administered a music-based intervention including self-paced movements of the index finger (MIDI-piano) and of the whole arm (drum pads), and they found that the music-supported therapy yielded significant improvement in both gross and fine motor functions of the hands; they speculated that the efficacy may be related to the external auditory feedback and neural reorganization induced by the melody and rhythm of music.^[53]^

### 4.7 Sensory-motor training in Parkinson's disease

Sensory input-based training is also a hot area of research in the rehabilitation of Parkinson's disease patients. For example, Taghizadeh et al have found that sensory-motor training for 2 weeks could improve both sensory performance (such as tactile acuity, wrist proprioception, and weight and texture discrimination) and upper extremity motor function in patients with Parkinson's disease; while these efficacies were limited to patients who had a score of 1 to 3 according to the Hoehn and Yahr Scale.^[54]^ Recently, nondrug treatments, especially music-based motor training, have been found to be effective for the motor functional rehabilitation of Parkinson's disease patients.^[47]^ Music can stimulate interactions between the sensory and motor systems, which may be helpful for evoking voluntary movements.^[55]^

### 4.8 Sensory input training on motor function recovery by interactions with neuroinflammation

Inflammation plays an important role in the pathogenesis of ischemic stroke and some metabolic diseases, and stroke represents an important central nervous system complication.^[56,57]^ Pretreatment with anti-inflammatory drugs for acute ischemic stroke may help patients achieve a favorable outcome.^[58]^ The sensory input training strategy may enhance motor rehabilitation through anti-apoptotic, neuroprotective, and anti-inflammatory effects.^[59]^

## 5 Conclusion

**Figure 2:**
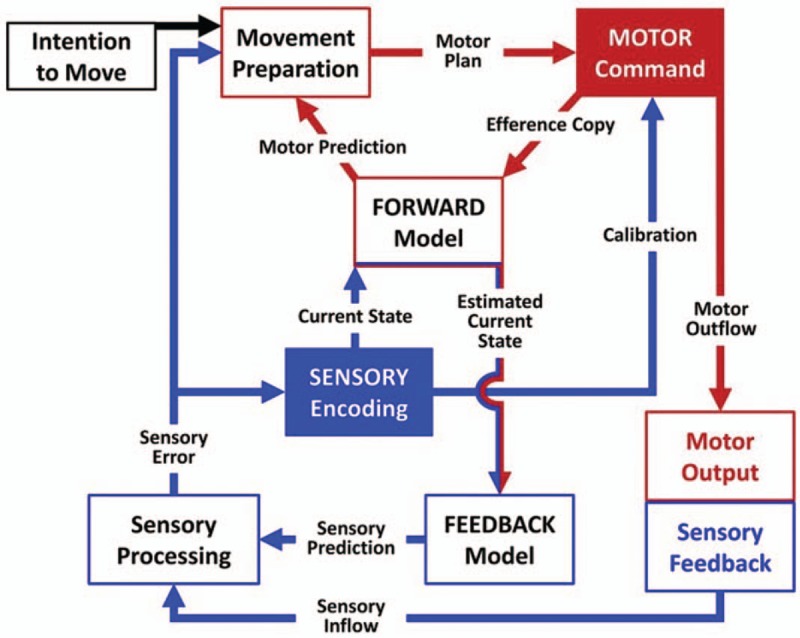
Sensory-motor integration circuits (reference ^[33]^).

In conclusion, sensory input plays a crucial role in motor rehabilitation (Fig. 2), and impairment of the sensory system can impact the motor functions. Therefore, sensory input should be highlighted in post-stroke rehabilitation. Our analysis indicates that a combined sensorimotor training modality is more effective than conventional motor-oriented approaches.

## Author contributions

**Data curation:** Xiaowei Chen, Zhaohong Yan, Xunchan Liu.

**Formal analysis:** Fuqian Liu, Shihuan Cheng, He Li.

**Funding acquisition:** Zhenlan Li.

**Investigation:** Shihuan Cheng, Xunchan Liu.

**Methodology:** Fuqian Liu.

**Resources:** Fuqian Liu.

**Validation:** Zhaohong Yan, He Li.

**Writing – original draft:** Xiaowei Chen.

**Writing – review & editing:** Zhenlan Li.
